# RaTexT®: a novel rapid tick exposure test for detecting acaricide resistance in *Rhipicephalus microplus* ticks in Brazil

**DOI:** 10.1186/s13071-024-06448-6

**Published:** 2024-08-28

**Authors:** Frans Jongejan, Laura Berger, José Reck, Priscila Teixeira Ferreira, Mariana Silveira de Jesus, Fabio Barbour Scott, Barbara Rauta de Avelar, Brena Gava Guimarães, Thais Ribeiro Correia, Dennis Muhanguzi, Patrick Vudriko, Joseph Byaruhanga, Maria Tumwebaze, Yakob Nagagi, Violet Temba, Abel S. Biguezoton, Souaïbou Farougou, Safiou Adehan, Humphrey Jumba, Laura Homminga, Iris Hulsebos, Alita Petersen, Guilherme Klafke

**Affiliations:** 1 Department of Veterinary Tropical Diseases, Faculty of Veterinary Science, University of Pretoria, Private Bag X04, Onderstepoort, 0110 Republic of South Africa; 2 TBD International BV, BioScience Center, Wageningen University Research, Runderweg 6, 8219 PK Lelystad, The Netherlands; 3 Instituto de Pesquisas Veterinárias Desidério Finamor, Estrada do Conde, 6000, Eldorado do Sul, RS 92990-000 Brazil; 4 Instituto de Veterinária da Universidade Federal Rural do Rio de Janeiro, Seropédica, Rio de Janeiro BR-465 Brazil; 5 College of Veterinary Medicine Animal Resources and Biosecurity, Makerere University, Kampala, Uganda; 6 Tanzania Plant Health and Pesticides Authority (TPHPA), P.O. Box 3024, Arusha, Tanzania; 7 Centre International de Recherche-Développement sur l’élevage en zone Subhumide (CIRDES) Bobo-Dioulasso, Bobo-Dioulasso, Burkina Faso; 8 Ecole Polytechnique d’Abomey-Calavi, Université Abomey-Calavi, Cotonou, Republic of Benin; 9 International Livestock Research Institute, P.O.Box 30709, Nairobi, 00100 Kenya

**Keywords:** Acaricide resistance, Larval Packet Test, Resistance Intensity Test, Rapid Tick exposure Test, *Rhipicephalus microplus*, Cattle ticks

## Abstract

**Background:**

Acaricide resistance in cattle ticks is a significant concern in (sub)tropical regions, particularly Brazil. The Larval Packet Test (LPT) is the standard laboratory bioassay for resistance diagnosis, which requires triplicates of seven acaricidal dilutions plus controls to cover larval mortalities ranging between 0 and 100%. The value of the LPT lies in providing resistance ratios based on the ratio between the LC50 calculated with potentially resistant and susceptible ticks. However, LC50 ratios are difficult to translate into practical advice for farmers. Moreover, LPT requires laboratory facilities to maintain susceptible tick colonies, and it takes 6 weeks to obtain the larvae to be tested by LPT derived from engorged female ticks collected from cattle in the field. Our novel approach was twofold: first, we upgraded the LPT to the Resistance Intensity Test (RIT) by adopting the latest WHO guidelines for resistance detection in mosquitoes, which combines a 1 × recommended dose with 5 × and 10 × concentrated doses to reveal low, moderate and high resistance intensity, respectively. This reduced the number of test papers and tick larvae and, more importantly, provided relevant information on the resistance level. Our second innovative step was to abolish testing larvae entirely and expose partly engorged adult ticks to the same acaricidal doses immediately after removing them from cattle in the field. This resulted in the Rapid Tick exposure Test (RaTexT®), wherein partly engorged adult ticks were exposed to an acaricide-impregnated, specially designed matrix providing test results within 24 h. This approach directly compared resistance detection in tick larvae in the RIT with resistance in adult ticks in RaTexT**®**.

**Methods:**

Laboratory validation was conducted in Brazil with resistant and susceptible colonies of *Rhipicephalus microplus* ticks. For field validation, adult *R. microplus* ticks collected from different cattle farms in Brazil were evaluated for resistance to RaTexT®, and the results regarding their larval progenies were compared with those for the RIT. Partly engorged adult ticks derived from cattle infested with laboratory and field strains of *R. microplus* were exposed to deltamethrin in RaTexT® containers, which contained six rows of four interconnected compartments, accommodating five to eight semi-engorged female ticks with a preferred size ranging between 5 and 8 mm. The corresponding larvae of each strain were exposed in the RIT to the same deltamethrin concentrations in filter papers.

**Results:**

In RaTexT®, mortality in adult ticks from a resistant strain of *R. microplus* from Seropédica in Brazil was 38.4%, 54.2% and 75.0% at the 1 ×, 5 × and 10 × doses of deltamethrin, respectively. In RIT, mortality of larvae from the same resistant strain was 2.0%, 4.9% and 19.5% at 1 ×, 5 × and 10 × doses, respectively. The results of RaTexT® and RIT agreed since both tests identified a high level of resistance based on a cut-off of 90% mortality.

In RaTexT®, mortality of adult ticks from a susceptible strain originating from Porto Alegre was 73.8%, 92.9% and 97.6% at the 1 ×, 5 × and 10 × doses, respectively. In RIT, mortality of larvae from the susceptible strain was 95.2%, 95.2% and 96.8% at the 1 ×, 5 × and 10 × doses, respectively. Interestingly, both tests identified a low number of unexpected resistant individuals in the susceptible strain since the mortality of neither larvae nor adults reached 100%. This effect remained unnoticed in the LPT, wherein a resistance ratio of 159.5 was found based on the LC50 of the resistant strain divided by the LC50 of the susceptible strain. Next, RaTexT® was compared with RIT using adult and larval ticks derived from three field strains of *R. microplus* in Brazil. RaTexT® detected high levels of resistance to deltamethrin in adult ticks in all strains, which was confirmed in larvae tested by the RIT. Both tests agreed on the same resistance level with significantly lower mortality rates in larvae than in adult ticks.

**Conclusions:**

RaTexT® is a novel rapid pen-site test for detecting acaricide resistance in adult livestock ticks. It potentially replaces laborious tests using larval ticks and provides results within 24 h relevant to acaricide resistance management of livestock ticks.

**Graphical Abstract:**

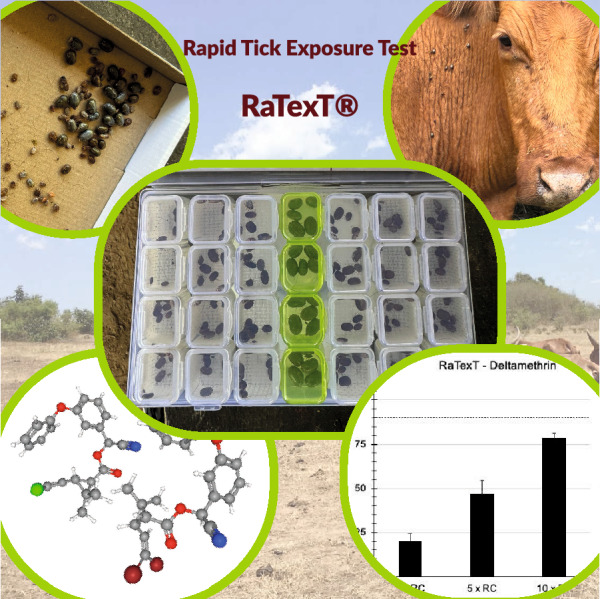

## Background

Ticks and tick-borne diseases rank high in their impact on animal and human health worldwide [1]. In livestock, tick-borne diseases caused by protozoan and rickettsial pathogens constitute a significant constraint to animal production, particularly in tropical and subtropical regions [2]. Tick control depends almost exclusively on applying acaricidal compounds to cattle and small ruminants [3–5]. However, resistant tick populations can be selected when repeatedly exposed to the same chemicals. As a result, acaricide resistance in ticks is a significant concern for the livestock industry in Latin America [4], sub-Saharan Africa [6, 7], parts of Asia [8] and Australia [5].

*Rhipicephalus* ticks are among the most damaging tick species on livestock. These ticks have diverse life cycles ranging from one to three host ticks. The one-host ticks develop resistance faster than any other tick species since they are submitted to selection in acaricide-treated cattle for almost three weeks. The main one-host cattle tick species is *Rhipicephalus microplus*, which builds up high tick burdens on livestock.

The situation in Brazil has been presented as a worst case scenario in the world, where resistance has been reported against six of seven classes of acaricides (i.e. organophosphates, formamidines, synthetic pyrethroids, macrocyclic lactones, phenylpyrazoles and benzoylphenyl ureas) [9, 10]. As tick control in Brazil is characterised by poor management practices [11], it will inevitably develop again against any novel class of acaricidal compounds [5]. With its vast pastures with large herds of susceptible cattle in a tropical environment, Brazil has ideal conditions for cattle ticks. Recently, *R. microplus* was shown to complete up to six generations annually in the Brazilian Amazonian region [12]. Resistance is highest in the primary cattle-producing states [9, 11]. Multi-resistant ticks have also been reported to occur elsewhere in Latin America [13].

Resistance has been reported against organophosphates, formamidines, synthetic pyrethroids, macrocyclic lactones, phenylpyrazoles and benzoylphenyl ureas [4–6, 14]. Resistance mechanisms include target-site mutations and increased metabolic detoxification. Tick resistance to acaricides is recognised as a failure of treatment to eliminate tick burdens from livestock. Although treatment failure often results from incorrect application of the acaricide or substandard acaricide products, the persistence of ticks after correctly applied treatments indicates that resistance has developed [15]. Although this article focuses on synthetic pyrethroids, RaTexT® can potentially be applied to other acaricidal classes. Therefore, a brief overview of all classes of acaricides is provided here.

Synthetic pyrethroids are sodium channel modulators that cause nerve excitation. They were introduced between 1975 and 1983 and include deltamethrin, (alpha) cypermethrin, flumethrin, permethrin and cyhalothrin. The pyrethroids have exceptional acaricidal properties and long residual effectiveness. Cypermethrin, deltamethrin, permethrin and cyhalothrin are mainly used to control ticks on livestock or companion animals. Resistance to synthetic pyrethroids in cattle ticks was first documented in the late 1980s in Brazil and Australia [4].

Organophosphates are acetylcholinesterase inhibitors introduced in the mid-1950s, including chlorpyrifos, chlorfenvinphos, diazinon and coumaphos. The first documented case of organophosphate resistance was in cattle ticks in Australia in the mid-1960s. Due to their relatively high toxicity to livestock, many products have been withdrawn from the market. However, they are still used in many combination products with active ingredients from other acaricidal classes. Formamidines (amitraz, cymiazole) exert toxicity on the octopamine receptor and were introduced for tick control in the mid-1970s. Amitraz is the most effective for controlling livestock ticks. Its toxicity to cattle and humans is minimal and less persistent. However, Australia's first amitraz resistance in ticks was reported in the early 1980s [3] and later in Brazil and Mexico [16]. Macrocyclic lactones were introduced in the mid-1970s to control various arthropods and nematodes. Doramectin, abamectin, eprinomectin and moxidectin have acaricidal properties and are increasingly used in combination products with other classes of active ingredients. The first report of resistance to ivermectin and doramectin in cattle ticks was in 2001 in Brazil and subsequently in Mexico, Argentina, Uruguay, India and South Africa [4]. Fipronil is the only phenylpyrazole compound used to control tick infestations on cattle. It was introduced in the mid-1990s. Resistance of *R. microplus* to fipronil has been reported in Brazil and Uruguay [17, 18]. Benzoylphenyl ureas are chitin synthesis inhibitors [19], and the first resistance against fluazuron was reported in *R. microplus* in 2014 in Brazil and 2022 in Argentina [10, 20].

Finally, isoxazolines are specific blockers of ligand-gated chloride channels and have dominated the companion animal ectoparasiticide market as oral canine products over the past decade [21, 22]. Importantly, though, the first isoxazoline-based commercial product (based on fluralaner) targeting ectoparasites on cattle has recently entered the livestock market in Brazil as a pour-on formula [23]. This is the first introduction of a novel class of acaricides in the livestock market in several decades. It was reported that applying fluralaner in summer and autumn, with 42-day intervals between treatments, effectively controlled *R. microplus* on cattle, which gained more weight than non-treated control cattle [24]. Although resistance to fluralaner has been selected by exposure of the house fly, *Musca domestica*, to this isoxazoline, there are no reports of resistance in ticks since the isoxazolines were introduced into the companion animal market in 2014 [25].

The Food and Agriculture Organization (FAO) recommends using the Larval Packet Test (LPT), Larval Immersion Test (LIT) and Adult Immersion Test (AIT) for resistance detection and monitoring in livestock ticks [26]. FAO initially referred to the LPT in its Plant Protection Bulletin, published in 1971, based on the original description by Stone and Haydock, published in 1962 [27]. The LPT Protocol was updated in the guidelines published in 2004 and has been widely adopted as an FAO reference test for detecting resistance in ticks [26]. The LIT protocol was modified by immersion of tick larvae inside microcentrifuge tubes [28–30]. AIT is a bioassay wherein engorged female ticks are immersed in a discriminating dose of an acaricide and subsequently monitored under laboratory conditions for their capacity to produce eggs. The more recently developed Larval Tarsal Test (LTT) employs a microtiter plate format [31]. It has been tested with field populations of *R. microplus* in Brazil [32] and evaluated with ticks from Argentina, South Africa and Australia [33]. However, despite all efforts, LTT has not become a mainstream test because of its complex equipment requirements.

The LPT has remained the primary test for detecting acaricide resistance since its introduction > 60 years ago [5]. However, drawbacks of the LPT are that the tests are laborious, need laboratory facilities and take at least 6 weeks before testing with larvae derived from engorged female ticks collected from animals in the field can be completed. Disputes between veterinary products suppliers and farmers about product efficacy are sometimes complex to settle since there is currently no rapid method to differentiate resistance from malpractices in chemical tick control. Therefore, FAO has recommended the development of a rapid pen-side bioassay to determine tick susceptibility or resistance to acaricidal products in a recent expert consultation [5].

In this article, we have upgraded the LPT to the Resistance Intensity Test (RIT) by adopting the resistance intensity protocol from the latest WHO guidelines for resistance detection in malaria mosquito vectors [34]. This protocol uses the recommended dose of the acaricide (1 ×), plus 5 × and 10 × higher concentrations, revealing low, moderate and high resistance intensity. This reduces the number of tests and provides relevant information on the resistance level at the farm and region.

Our second innovation was to abolish testing larvae entirely and expose semi-engorged adult ticks to acaricides immediately after removing them from cattle in the field. This article presents the proof of principle that partly engorged adult ticks can rapidly diagnose acaricide resistance in ticks by confining them in small compartments exposed to an acaricide-impregnated, specially designed matrix.

## Methods

### Tick strains

The susceptible reference colony of *R. microplus* originated from a ranch in Palmares do Sul, Rio Grande do Sul State, Brazil, in 1992. It has been maintained on cattle at the Universidade Federal do Rio Grande do Sul facilities in Porto Alegre and designated Porto Alegre. The resistance laboratory reference strain, designated RBm UFRRJ, originated from Seropédica in Brazil and has been maintained on cattle since 2018. RBm UFRRJ is resistant to deltamethrin, cypermethrin, chlorpyriphos, chlorfenvinphos, amitraz, doramectin, ivermectin and fipronil (data not shown). Field isolates of *R. microplus* were collected from cattle farms in Eldorado do Sul (RBm01), Guaíba (RBm02) and Butiá (RBm03).

Engorged female ticks were collected from cattle and incubated in 150-mm glass rearing vials closed with a stopper, which allowed ventilation. These vials were placed in an incubator at 28 °C, with relative humidity between 85 and 95%. Larvae of 2 to 4 weeks age were used for the experiments.

### Preparation of ticks

The preparation of ticks followed the recommendations of FAO (2004). For the larval tests (LPT and LIT) with Porto Alegre and RBm UFRRJ strains and field isolates RBm01, 02 and 03, at least 60 fully engorged female ticks were manually collected from cattle, washed with water and dried with paper towels. Groups of 20 ticks were allocated in 90-mm-diameter × 10-mm plastic Petri dishes. The Petri dishes were kept at 28º C ± 1 ºC and 85% ± 5% relative humidity in an incubator for 14 days to allow the ticks to lay eggs. After 14 days, the egg masses were gently mixed with a metal spatula and separated in aliquots of 500 mg each inside serum vials (5 ml capacity) that were closed with cotton plugs. The vials were icubated at the same conditions for another 4 weeks to allow larvae to hatch. The experiments were performed between 4 to 6 weeks after the incubation of the eggs (i.e. larvae with 14 to 28 days of age, approximately).

For the Rapid Tick exposure test, female ticks between 5 and 8 mm in size were carefully removed from the hosts to avoid damaging their mouthparts. The ticks were used immediately in the experiments.

### Manufacturing of the Rapid Tick exposure Test

The RaTexT matrix was impregnated with commercial acaricide formulations at the manufacturer's recommended concentration, plus 5 × and 10 × higher concentrations derived from 5% deltamethrin (Vectocid®) (Ceva Animal Health, Libourne, France). The concentrations of deltamethrin were 0.25 mg/ml, 1.25 mg/ml and 2.5 mg/ml for the 1 ×, 5 × and 10 × concentrated doses, respectively. The nylon matrix was cut into the required format using a laser-driven ScanNCut (Zijlstra, Groningen, The Netherlands) and impregnated in a separate zip bag for each dilution. The total amount of deltamethrin was calculated and diluted in a mixture of trichloroethylene with olive oil (2:1). One piece of the matrix was impregnated with 0.37 ml diluent. The control matrix was first impregnated using trichloroethylene/olive oil diluent. Each acaricide mixture was adequately mixed with the matrix pieces inside the zip bag to ensure a homogeneous coating. Afterwards, the matrix pieces were thoroughly dried in an aluminium tray in a fume hood overnight. Each piece of matrix was fixed to the bottom of each compartment of the RaTexT® container with a tiny drop of RTV-1 elastosil E4 silicon glue on the compartment's bottom. The inside of the lid remained uncovered.

### Rapid Tick exposure Test (RaTexT®)

Partly engorged *R. microplus* ticks were manually removed from cattle and collected into a plastic beaker, which was subsequently emptied into a shallow cardboard box where they were allowed to move around and selected based on sex, vitality (actively walking) and preferred size. Excess moisture or dirt was left behind in the box, eliminating the need for washing since ticks must be dry when inserted into RaTexT**®**. Depending on the availability, five to eight partly engorged female ticks were inserted into each compartment with a pair of tweezers, starting with the control and ending with the highest acaricide concentration (10 ×). Then, all lids were firmly closed, and the RaTexT® container was placed inside a plastic zip bag with a piece of moist tissue and sealed completely. Each container was left at room temperature and out of direct sunlight for 24 h. After 24 h, ticks were removed from the compartments, starting with the controls to record the number of live and dead ticks. The number of knocked-down ticks was recorded in the Data Capture Form. A tick was considered dead or ‘knocked down’ if it had lost its ability to walk. This procedure was facilitated by placing each tick inside a small grey circle on the Data Capture Form. Ticks that could walk out of the circle were recorded as alive versus those that could not move even after stimulating them with breath and gently prodding them with blunt forceps. After counting, all ticks were discarded inside a plastic zip bag containing ethanol, and forceps were cleaned with ethanol after usage. Each row of four interconnected compartments was considered a replicate, and experiments were replicated six times for each tick strain.

### Resistance Intensity Test (RIT)

The Resistance Intensity Test was impregnated with a commercial deltamethrin (Vectocid®) formulation at the manufacturer's recommended concentration, plus 5 × and 10 × higher concentrations [35]. The concentrations of deltamethrin were 0.25 mg/ml, 1.25 mg/ml and 2.5 mg/ml for the 1 ×, 5 × and 10 × concentrated doses, respectively. Dilutions were made in triplicate and impregnated into 10 × 7-cm Whatman (no. 1) quality filter papers (Merck Life Science, Amsterdam). RIT filter papers were impregnated simultaneously with the RaTexT matrices using the same diluted acaricidal concentrations for optimal comparison. The control matrix was first impregnated using trichloroethylene/olive oil diluent. Then, the filter papers were thoroughly dried in an aluminium tray in a fume hood for at least 60 min. The filter papers were subsequently grouped by concentration and sealed in plastic bags, which were were kept in the dark at room temteraturte until used.

For the test, each filter paper was folded in width to create a packet and sealed by bulldog clips [26]. A tick-proof working station was loaded with water containing a 1% cleaning/disinfectant and double-sided tape fitted around the edge of the working station as an additional barrier. Approximately 50 larvae were taken from the rearing tubes and added to each packet using a fine brush. Control packets were loaded first, and the packets with the highest acaricide concentration were filled last. The sealed larval packets were placed in a container wherein a saturated atmosphere of potassium sulphate was created (approximately 85% relative humidity). Incubation was at room temperature for 24 h. After that, each packet was opened, and both live and dead larvae were counted, starting with the control and working up to the highest concentration. After counting, all packets with dead and alive ticks were disposed of into the water reservoir, ethanol was added, and they were kept close to the working station with the lid. The criterion for mortality was the larvae's inability to walk after CO_2_ stimulation by gently breathing after the packets had been opened. In each test, three packets of each concentration were loaded with larvae. Three independent tests were carried out with the reference strains (Posto Alegre and RBm UFRRJ). One complete test was performed with each field isolate.

### Larval Packet Test (LPT)

LPT was carried out as described before [26]. Briefly, six serial dilutions of deltamethrin (i.e. 0.015; 0.032, 0.15, 0.25, 1.25 and 2.5 mg/ml) were impregnated into 7 × 10-cm Whatman quality (no. 1) filter papers (Merck Life Science, Amsterdam) in triplicate using a mixture of olive oil and trichloroethylene as the solvent. The control filter papers were impregnated only with trichloroethylene and olive oil (2:1). Thereafter, the filter papers were grouped according to concentration and sealed in plastic bags which were kept in the dark at room temperature until used.

At the moment of testing, each filter paper was folded in width to create a packet, sealed by bulldog clips [26]. Between 50 and 100 larvae were removed from the rearing tube added to each packet using a fine brush. The packets were incubated at room temperature for 24 h in a desiccator, wherein a saturated atmosphere of potassium sulphate was created (85% relative humidity). After that, live and dead larvae were counted, starting with the control packets. Test replicates were performed as described above for the RIT.

### Statistical analysis

Two-sample t-tests (*P* < 0.005) were used to test for significant differences between the mortality of ticks in the resistant *Rhipicephalus* strain versus mortality in the susceptible reference strain for each concentration. The percentage mortality of adult ticks recorded in the RaTexT**®** was compared with that of larval ticks found in the RIT. The results were statistically analysed using the two-proportion *Z*-test based on a normally distributed dataset. For this purpose, the two-proportion Z-test required a minimal sample size of 30 adult ticks per concentration. The following null hypothesis was tested *H*_0_: *μ*_1_ = *μ*_2_ (wherein both population proportions are equal).

The following formula was used to calculate the statistic *z* value:

$Z-value=\frac{{{\varvec{p}}}_{1}-{{\varvec{p}}}_{2}}{\sqrt{{\varvec{P}}(1-{\varvec{P}})(\frac{1}{{{\varvec{n}}}_{1}}+\frac{1}{{{\varvec{n}}}_{2}})}}$ where p_1_ and p_2_ are the sample proportions, n_1_ and n_2_ are the sample sizes, and *p* is the total pooled proportion calculated as $P=\frac{{p}_{1}+{p}_{2}}{{n}_{1}+{n}_{2}}$.

The *P*-value was calculated from the Z-value with the Z table. The null hypothesis was rejected if the *P*-value corresponding to the test statistic Z was less than the chosen significance level (*P* < 0.01).

Finally, Abbott’s formula was used to correct for mortality of 10% or less:

$$Corrected\,mortality\,percentage\,= \,\frac{\%\,test\,mortality\,-\,\%\,control\,mortality}{100\,-\,\%\,control\,mortality}\,\bullet 100$$

A 90% mortality cut-off was used based on the WAAVP guidelines recommendation for acaricide efficacy in the field. The decision tree was used to determine whether ticks were susceptible or carried a low, medium or high resistance level (Table 1).

**Table 1:** Decision tree (step 1–4) to determine the resistance level of ticks exposed to different concentrations of acaricides in RaTexT® and RIT

1	A	Mortality in control ≤ 10%	Use Abbott’s formula for correction 2
	B	Mortality in control > 10%	Disregard this test and start again
2	A	Mortality at 1 × recommended dose ≥ 90%	**Susceptible**
	B	Mortality at 1 × recommended dose < 90%	3
3	A	Mortality at 5 × concentrated dose ≥ 90%	**Low resistance**
	B	Mortality at 5 × concentrated dose < 90%	4
4	A	Mortality at 10 × concentrated dose ≥ 90%	**Moderate resistance**
	B	Mortality at 10 × concentrated dose < 90%	**High resistance**

For the LPT, a probit analysis was performed on the mortality results using Polo-Plus (1.0, LeOra Software) software to generate the dose-response plots. The following parameters were determined for each test: lethal concentrations for 50% (LC50), confidence intervals of 95% (CI 95%) and the slope of the regression line. Resistance ratios (RR) and their CI of 95% were generated with the software Polo-Plus using the formula described by Robertson et al. [36]. The significance of each comparison was determined when the calculated confidence intervals did not overlap.

## Results

**Fig. 1:**
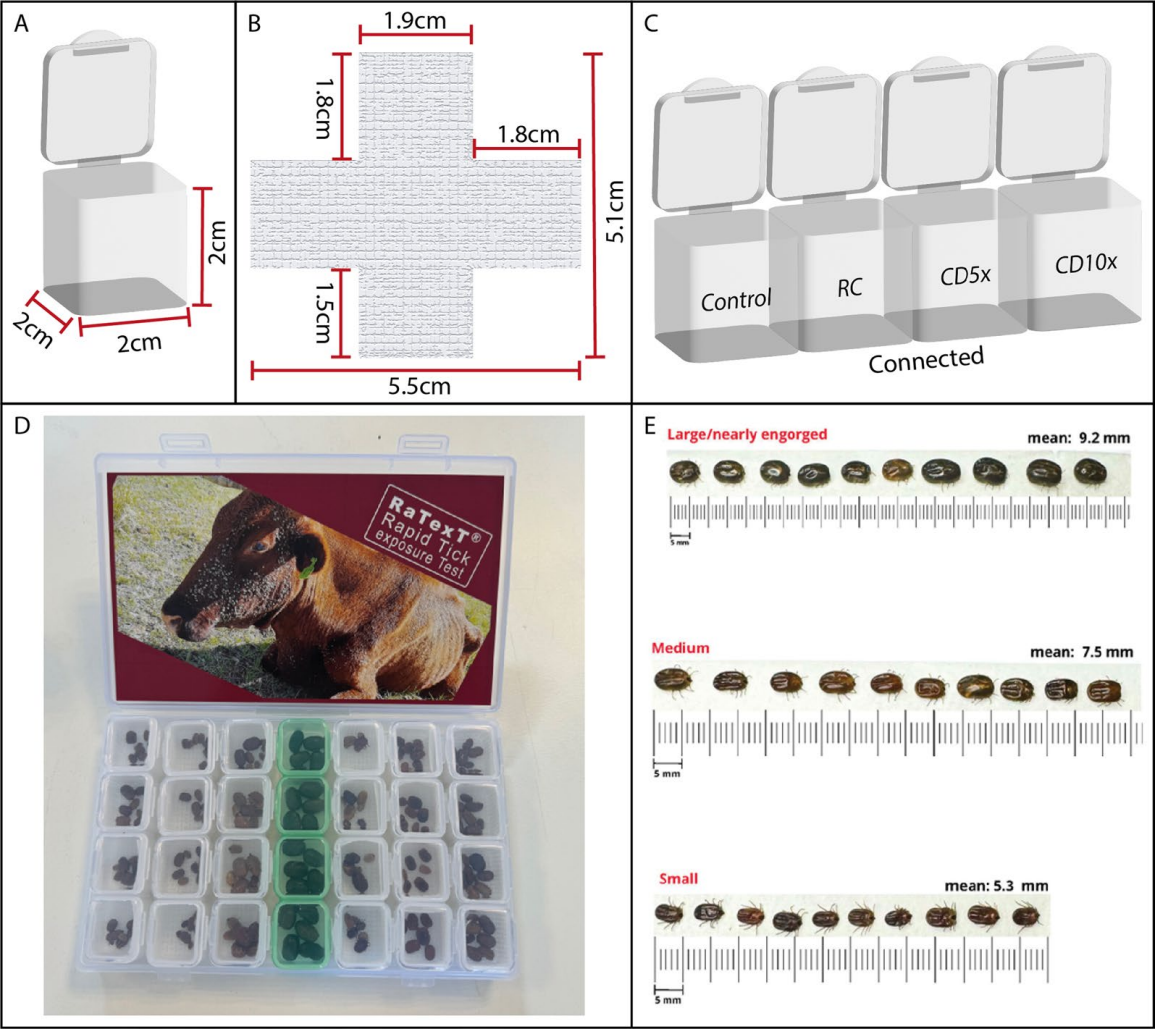
The Rapid Tick exposure Test (RaTexT®) design consists of small compartments (**A**) fitted with an impregnated matrix (**B**) in rows of four interconnected compartments (**C**). Each RaTexT box is filled with semi-engorged ticks in rows 1–3 and 5–7, whereas row 4 (in green) contains fully engorged ticks for further comparative studies (**D**). The preferred sizes of partly engorged *Rhipicephalus microplus* ticks for inclusion into RaTexT® are illustrated in panel **E**

Partly engorged adult ticks derived from cattle infested with laboratory and field strains of *R. microplus* were confined to small compartments (Fig. 1a) lined with a specially designed matrix (Fig. 1b) and impregnated with recommended concentrations of deltamethrin (0.25 mg/ml), 5 × concentrated doses (1.25 mg/ml) and 10 × concentrated dose (2.5 mg/ml) and untreated controls (Fig. 1c). Each RaTexT® box contained six rows of four interconnected compartments (Fig. 1d). Each compartment accommodated at least five semi-engorged female ticks with a preferred size ranging between 5 and 8 mm with a total number of 120 ticks per box (Fig. 1e). The untreated central row (in green) carried fully engorged females from the same animals back to the laboratory for subsequent testing of their corresponding larval progeny (Fig. 1d).

**Fig. 2:**
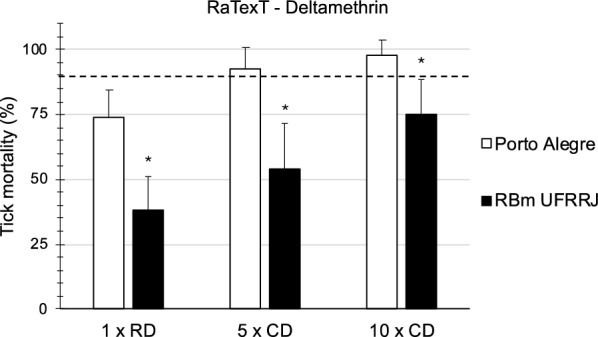
Comparison of a susceptible strain (Porto Alegre) with resistant strain (RBm UFRRJ) of *Rhipicephalus microplus* semi-engorged adult females in the RaTexT® against deltamethrin. RD: recommended dose. CD: concentrated dose. Error bars represent the standard deviations. The dashed line represents the 90% mortality cut-off value. Asterisks indicate significant differences (two sample t-tests; *P* < 0.005) in the mortality of the susceptible reference strain in the same concentration

**Fig. 3:**
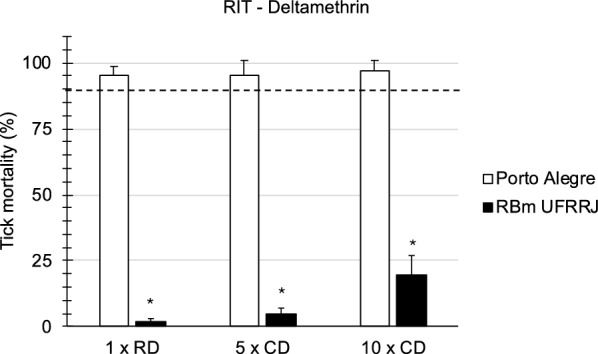
Comparison of a susceptible strain (Porto Alegre) with a resistant strain (RBm UFRRJ) of *Rhipicephalus microplus* larvae in the Resistance Intensity Test (RIT) against deltamethrin. RD: recommended dose. CD: concentrated dose. Error bars represent the standard deviations. The dashed line represents the 90% mortality cut-off value. Asterisks indicate a significant difference (two sample t-tests; *P* < 0.001) in the mortality of the susceptible reference strain in the same concentration

In the first series of experiments, the Rapid Tick exposure Test using adult ticks was compared with the Resistance Intensity Test using larval batches of ticks derived from laboratory strains of *R. microplus*. A resistant laboratory strain from Seropédica in Brazil (designated RBm UFRRJ) was compared with a susceptible laboratory strain of the same tick species originating from Porto Alegre (Fig. 2). In RaTexT®, mortality in adult ticks from a resistant strain of *R.microplus* from Seropédica in Brazil was 38.4%, 54.2% and 75.0% at the 1 ×, 5 × and 10 × doses of deltamethrin, respectively (Fig. 2). In RIT, mortality of larvae from the same resistant strain was 2.0%, 4.9% and 19.5% at 1 ×, 5 × and 10 × doses, respectively (Fig. 3). Both tests identified a high level of resistance according to a decision tree with a cut-off of 90% mortality (Table 1). The two-proportion Z-test null hypothesis was rejected as mortality percentages in RaTexT® and RIT were unequal (Table 2).

**Table 2:** Comparison of the percentage mortality in RaTexT® and RIT with the two-proportions Z-test combined with the conclusions derived from the decision tree

Strain	RD1x *P*-value (Z-value)	CD5x *P*-value (Z-value)	CD10x *P*-value (Z-value)	Conclusion	
				RaTexT	RIT
Porto Alegre	0.129 (1.518)	0.709 (0.373)	0.919 (0.101)	Low resistance	Susceptible
RBm UFRRJ	< 0.0001 (− 40.171)	< 0.0001 (− 33.176)	< 0.0001 (− 9.571)	High resistance	High resistance
RBm01	< 0.0001 (− 45.436)	< 0.0001 (− 30.334)	< 0.0001 (− 24.318)	High resistance	High resistance
RBm02	< 0.0001 (− 24.128)	< 0.0001 (− 25.752)	< 0.0001 (− 20.939)	High resistance	High resistance
RBm03	< 0.0001 (− 4.267)	0.269 (− 1.105)	< 0.0001 (− 7.914)	High resistance	High resistance

**Fig. 4:**
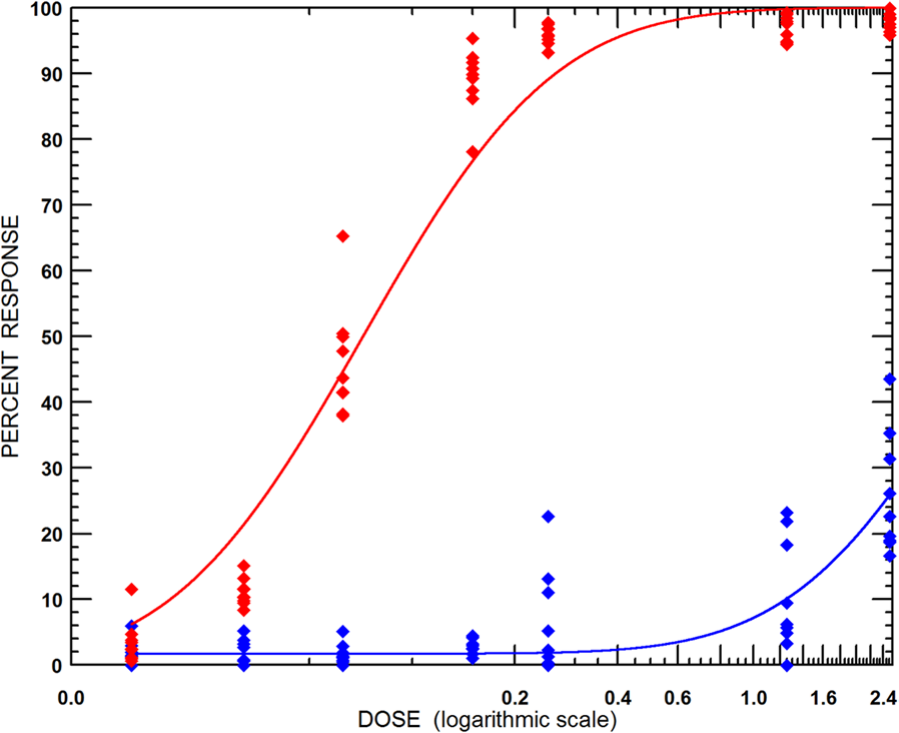
Comparison of a susceptible strain (Porto Alegre—red line and dots) with a resistant strain (RBm UFRRJ—blue line and dots) of *Rhipicephalus microplus* larvae with the Larval Packet Test (LPT) against deltamethrin

In RaTexT®, mortality of adult ticks from the susceptible strain (Porto Alegre) was 73.8%, 92.9% and 97.6% at the 1 ×, 5 × and 10 × doses, respectively (Fig. 2). In RIT, mortality of larvae from the susceptible strain was 95.2%, 95.2% and 96.8% at the 1 ×, 5 × and 10 × doses, respectively (Fig. 3). Both tests identified a low number of unexpected resistant individuals in the susceptible strain since the mortality of larvae and adults was < 100%. This effect remained unnoticed in the LPT, wherein a resistance ratio of 159.5 was found based on the LC50 of the resistant strain divided by the LC50 of the susceptible strain (Fig. 4).

**Fig. 5:**
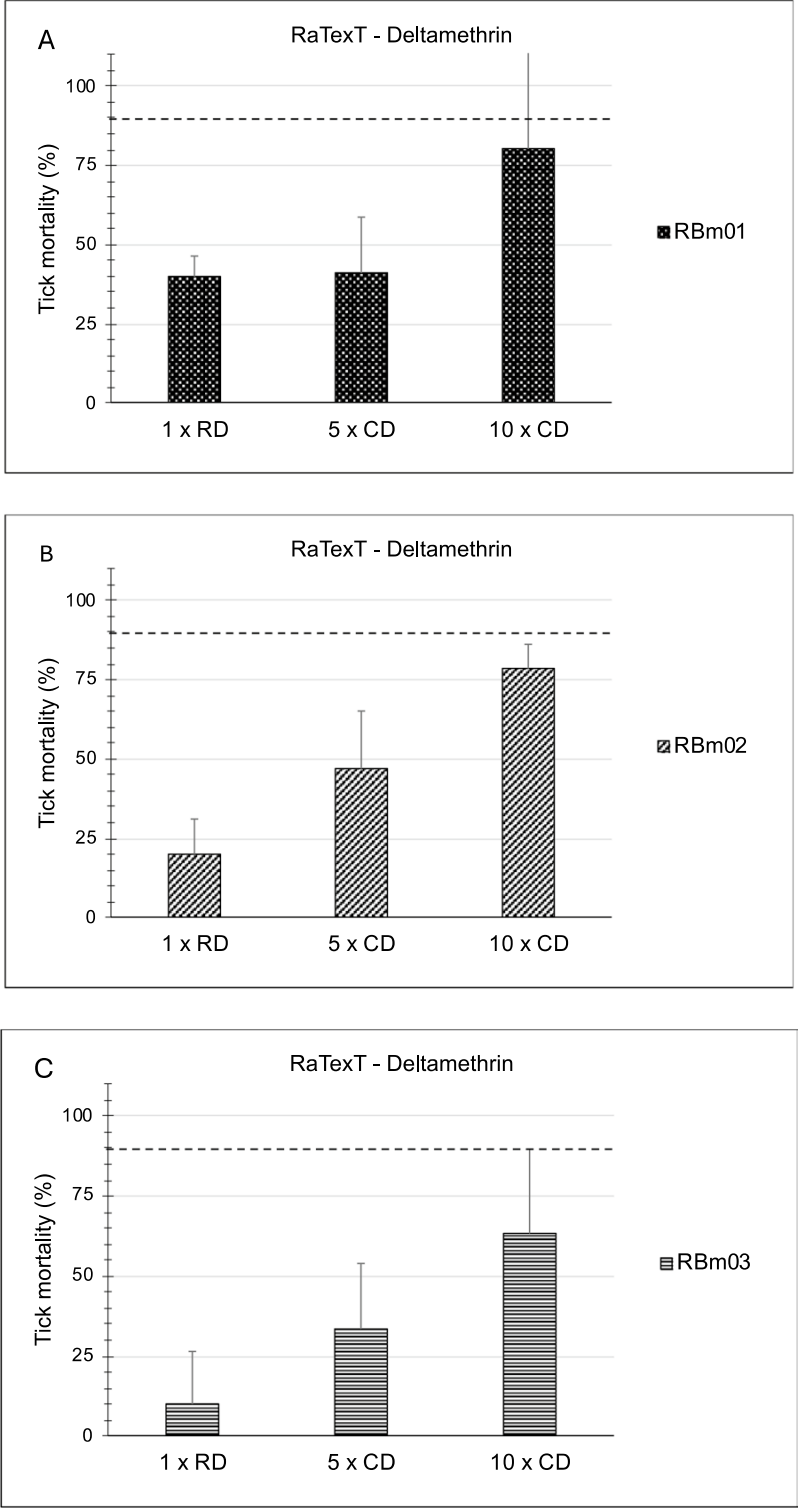
RaTexT® against deltamethrin with Brazilian field strains of *Rhipicephalus microplus* semi-engorged adult females. **A** (RBm01), (**B**) (RBm02) and (**C**) (RBm03) are highly resistant to deltamethrin. RD: recommended dose. CD: concentrated dose. Error bars represent the standard deviations. The dashed line represents the 90% mortality cutoff value

**Fig. 6:**
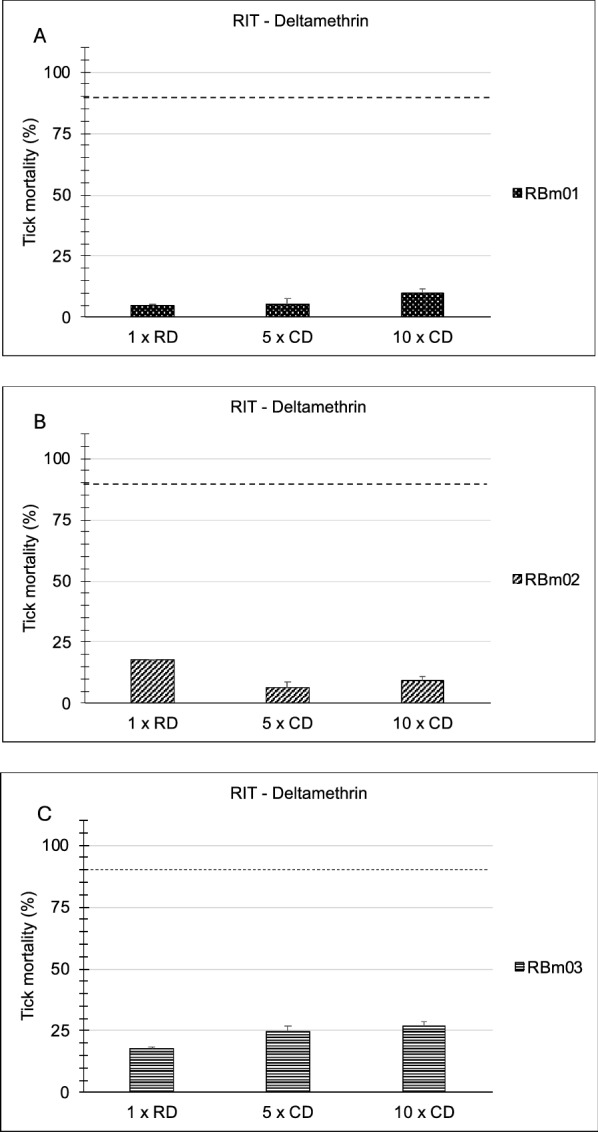
Resistance intensity test against deltamethrin with Brazilian field strains of *Rhipicephalus microplus* larvae. **A** (RBm01), (**B**) (RBm02) and (**C**) (RBm03) are highly resistant to deltamethrin. RD: recommended dose. CD: concentrated dose. Error bars represent the standard deviations. The dashed line represents the 90% mortality cutoff value

**Fig. 7:**
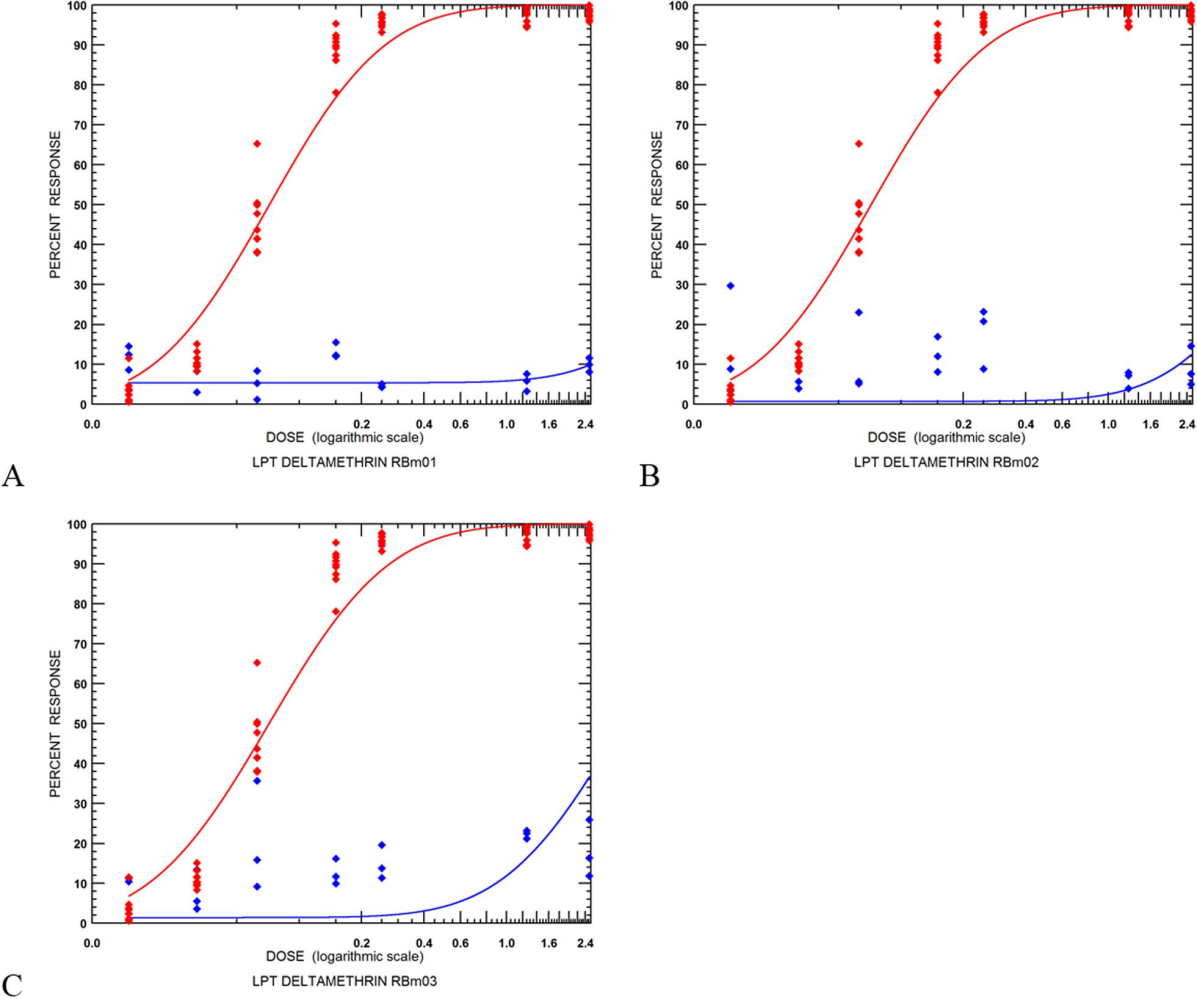
Dose–response plots of field strains of *Rhipicephalus microplus* submitted to the Larval Packet Test with deltamethrin. Porto Alegre—red line and dots; RBm01 (**A**), RBm02 (**B**) and RBm03 (**C**)—blue line and dots

In the second series of experiments, RaTexT® was compared with the RIT using adult ticks and tick larvae derived from field strains collected in Brazil. The three field strains originated from cattle at farms situated in Eldorado do Sul (RBm01), Guaíba (RBm02) and Butiá (RBm03) in Rio Grande do Sul, Brazil. RaTexT® detected a high level of resistance to deltamethrin in adult ticks (Fig. 5), which was confirmed by testing larvae in the RIT (Fig. 6). Notably, in general, mortality rates in larvae were lower than in the adult ticks (Figs. 5, 6). Both tests identified a high resistance level according to a decision tree with a cutoff of 90% mortality (Table 1). The two-proportion Z-test null hypothesis was rejected as mortality percentages in RaTexT® and RIT were unequal (Table 2). Finally, the LC50 based on the resistance ratio of resistant strains compared with a susceptible strain with the Larval Packet Test (LPT) against deltamethrin resistance appeared out of the normal range of LPT (highest dose 2.5 mg/ml), and LC50 could not be calculated (Fig. 7).

## Discussion

RaTexT® is a significant innovation since Stone and Haydock introduced the Larval Packet Test over 60 years ago [27]. This article presents the proof of principle of RaTexT® as a novel rapid tick exposure test for detecting resistance to acaricides in livestock ticks. The test consists of a transparent polypropylene container with six rows of four small, interconnected compartments, wherein adult ticks are exposed to an acaricide-impregnated matrix immediately after being removed from cattle in the field (Fig. 1). Five to eight medium-sized semi-engorged female ticks between 5 and 8 mm were the preferred size of *R. microplus* ticks (Fig. 1). Smaller ticks are more vulnerable, whereas the nearly fully engorged ticks do not fit into the compartments of the RaTexT® container.

It is not the first time adult ticks have been used to detect acaricide resistance in livestock ticks. Drummond et al. described the Adult Immersion Test (AIT) in 1973 [37] and recommended it as a preliminary screening test for resistance. In this test, engorged female ticks are immersed in a discriminating dose of an acaricide and monitored under laboratory conditions for their capacity to produce eggs. However, an inter-laboratory study failed to identify a standard discriminating dose for the Adult Immersion Test [38]. This required alternative ways to make a meaningful comparison between RaTexT® and existing acaricide resistance tests.

Before conducting the experiments in Brazil, laboratory colonies at the Instituto de Pesquisas Veterinárias Desidério Finamor in Eldorado do Sul were tested against deltamethrin with the LPT, which revealed a resistance factor of 159.5 (Fig. 4). Although this provided the basis for introducing RaTexT®, which differentiated deltamethrin-resistant *R. microplus* ticks from susceptible *Rhipicephalus* ticks (Fig. 2), it was realised that a direct comparison with LPT was not the best way forward. LPT is based on resistance factors: the ratio of an LC50 of a resistance strain and a susceptible reference strain. In addition to the time required to produce LPT results, once available, it sometimes adds little value to solving an acaricide resistance problem in the field. Another approach with LPT has been to use a discriminating dose, reducing the number of test dilutions to only one concentration, whereby 100% of susceptible larvae are killed. This approach has two limitations: (1) since a technical grade acaricidal compound is often used for this purpose, a discriminating dose may not correspond with the formulated product on the market; (2) there is no universal discriminating dose of any of the acaricidal classes because of regional differences in susceptibility.

The solution was to upgrade the LPT and create the Resistance Intensity Test (RIT) by adopting the latest WHO guidelines for resistance detection in mosquitoes, which combines a 1 × recommended dose with 5 × and 10 × concentrated doses to reveal low, moderate and high resistance intensity, respectively [34, 35]. In addition to reducing the number of test papers and larval ticks, using the recommended concentration of an acaricide for impregnating filter papers created a direct link to acaricidal products available on the market. Moreover, by adding a 5 × and 10 × concentrated dose, the RIT also provided relevant information on the resistance level in livestock ticks. Importantly, the same 1 × recommended dose with 5 × and 10 × concentrated doses were also impregnated into RaTexT®. This created a direct comparison between the detection of resistance in tick larvae in an upgraded LPT and the detection of resistance in adult ticks in the novel Rapid Tick exposure Test (RaTexT®). It was envisaged that, through both resistance intensity assays, it would be possible to identify areas where resistance is most intensively expressed and where a re-assessment of acaricide management strategies would make a difference in recent studies conducted in Brazil and elsewhere[39, 40].

The comparison of RaTexT® with RIT in Brazil revealed that both tests detected resistance to deltamethrin in laboratory colonies (Figs. 2, 3) and field strains of *R. microplus* (Figs. 5, 6). According to the decision tree, both tests agreed concerning the resistance level: high-intensity resistance based on a 90% mortality cutoff value (Table 1). The two-proportion Z-test null hypothesis of equality was rejected because of the significantly lower mortality rates of larvae in RIT than in adult ticks in RaTexT® throughout the experiments (Figs. 2, 6 and Table 2). Whether this indicated that adults were more sensitive than larvae or only exposed to a larger acaricidal dose remains to be shown. This needs to be further studied by conducting more comparative tests, which is the limitation of the current article, wherein the proof of principle of test agreement is presented based on a limited dataset. A further interesting observation was that both tests identified a low number of unexpected resistant individuals in the susceptible strain since the mortality of larvae and adults remained < 100%. When engorged females derived from this susceptible strain were exposed to synthetic pyrethroids in the AIT, some could produce eggs (unpublished obversations). A preliminary molecular analysis revealed the presence of low-frequency Kdr mutations in the sodium channel of these ticks (unpublished information). Notably, the Porto Alegre strain, isolated from a field population that might have been exposed and selected for SP resistance before the establishment of the colony in 1992, and a few resistant individuals are maintained in low frequency in the lineage in the absence of selection. We can observe the presence of these individuals when exposing the ticks to the therapeutic dose of deltamethrin, or even at 5 × or 10 × concentrated doses.

RaTexT**®** is simple to operate, provides rapid results and has no drawbacks linked to the other acaricide resistance tests. This method significantly enhances efficiency and productivity in acaricide resistance testing and does not require a susceptible reference strain. Here, the proof of principle of using adult ticks in a rapid exposure bioassay is illustrated with deltamethrin. Moreover, we have used RaTexT® impregnated with other classes of acaricides and identified resistance against fipronil and chlorpyrifos in *R. microplus* ticks in Brazil (unpublished observations). RaTexT® can be used with a broad range of ticks species derived from livestock or companion animals. Finally, further evaluation of RaTexT® is currently being conducted in East Africa, where resistance to synthetic pyrethroids was identified in adult *Rhipicephalus decoloratus* and *Rhipicephalus appendiculatus* ticks within 24 h after removing them from cattle in the field.

## Data Availability

The whole dataset used in this study is available upon request. RaTexT**®** is a registered trademark patented under registration number 2036918. RaTexT**®** is manufactured and distributed by TBD-I. No datasets were generated or analysed during the current study.

## Abbreviations

LPT: Larval Packet Test
RIT: Resistance Intensity Test
RaTexT: Rapid Tick exposure Test
